# The evidence—and acceptability—of taxes on unhealthy foods

**DOI:** 10.1186/s13584-018-0264-6

**Published:** 2018-11-20

**Authors:** Selvi Rajagopal, Anne Barnhill, Joshua M. Sharfstein

**Affiliations:** 1 General Preventive Medicine Residency, Johns Hopkins Bloomberg School of Public Health, Baltimore, USA; 2 Berman Institute of Bioethics, Johns Hopkins University, Baltimore, USA; 3 Department of Health Policy and Management, Johns Hopkins Bloomberg School of Public Health, 615 N. Wolfe Street, W1033F, Baltimore, MD 21205 USA

**Keywords:** Unhealthy food and beverage taxes, Policy, Ethics, Public health, Obesity, Food industry, Lower socioeconomic status

## Abstract

The global obesity pandemic has public advocates and policymakers grappling with the question of how best to respond. Among the various policy options, unhealthy food and beverage taxes have gained attention as a potentially effective intervention to reduce non-nutritive caloric intake, while raising government funds for health promotion programs at the community level. Yet in many countries, including in Israel, such proposals have not gained broad support. Cities in both United States and Mexico have found that taxes on sugar-sweetened beverages reduce consumption. Yet the food industry has successfully fought many such policies. Looking forward, those supporting taxation policies will need to provide clear evidence, a compelling use of funds raised, a convincing answer to industry claims, and attention to equity in implementation. With no easy fixes in sight to obesity, it is likely that taxes will remain viable – if contested – options for the foreseeable future.

With more than a quarter of global mortality now attributed to diseases related to obesity, including cardiovascular disease and stroke [1], it is no surprise that policymakers are considering a range of potential responses. Of growing interest are proposals that would increase the price of unhealthy foods in order to lower their consumption. Advocates for these policies often cite as a model the experience of cigarette taxes, which have been established as effective and widely adopted to reduce tobacco-related disease [2].

Tamir et al. recently published a qualitative assessment of key stakeholders’ views on taxes on unhealthy food and beverages in Israel [3]. The researchers found that a broad range of interested persons -- including legislators, healthcare providers, parents and families -- agreed that obesity was a problem of significant public concern. However, few believed that taxes on unhealthy food and beverage would lead to lower consumption. Moreover, legislators appeared particularly skeptical that such taxes would be acceptable to Israelis.

This research introduces two important questions: Do taxes on unhealthy foods improve the diet? And if so, why is there not more support for imposing them?

Evidence on effectiveness comes primarily from experience in the United States and Mexico [4]. U.S. jurisdictions that have a tax on sugar sweetened beverages (SSBs), include four in California (Albany, Oakland, Berkeley, and San Francisco), the cities of Seattle, Boulder, and Philadelphia, and the Navajo Nation. The first city to adopt the SSB tax was Berkeley, California in 2014, with a $0.01 per ounce excise tax on SSBs, of which an estimated 47% of the tax was transferred to retail customers through sales prices [5]. Within 4 months of policy implementation, the city saw a 21% reduction in the consumption of SSBs and a 68% increase in the consumption of water. These changes were concentrated in lower socioeconomic communities, where the burden of obesity is highest [5]. Philadelphia observed similar results following its tax legislation [6]. Mexico’s 2014 excise tax on SSBs yielded a 5.5% reduction in the purchase of taxed beverages over the first year and a 9.7% reduction in the second year, with the largest decrease in purchases observed among lower socioeconomic households [7]. Mexico’s 8% sales tax on unhealthy foods also resulted in a 5.8% decline in the purchase of taxed foods among households of medium socioeconomic status households, and a 10.2% decline among households of lower socioeconomic status [8]. It is too early to assess the impact of these taxes in the context of market and policy changes on overall dietary change and obesity rates.

With clear evidence that taxes reduce consumption of unhealthy food and beverages, why is their adoption so contested? In part, the controversy reflects a sustained campaign of opposition by the food industry, including manufacturers of SSBs. A primary message of this campaign is that taxation is an undue restriction of individual choice and is unfair towards low-income families. Lobbying groups echo the message of the food industry [9, 10] and frame taxes as an unwarranted violation of Americans’ freedom, with one website proclaiming “all of us are tired of the government trying to control us.” [10] A survey study of Americans’ opinions of policies aimed to reduce SSB consumption found that 65% of people responded favorably for calorie labeling, which would afford greater choice to the consumer, but 76% opposed SSB taxes [11]. Most recently, following intense lobbying from the American Beverage Association, California imposed a ban on further soda taxes until 2031 [12].

Whether advocates of taxes on unhealthy food can overcome opposition depends on their ability to develop and disseminate a compelling case. In addition to the evolution of the scientific evidence, two factors may play an outsized role in the outcome of policy debates.

The first factor is how the revenue raised from taxes will be spent. As a public health intervention alone, a tax on unhealthy food may not be broadly appealing. However, in all US cities where the tax is employed, millions of dollars in revenue are being collected by local governments, who have earmarked funds for health promotion in low income communities, through health education, parks and recreation development, improved drinking water access, pre-kindergarten education, and chronic disease prevention. Philadelphia used the $72 million in tax revenue to send 2400 children to pre-school [13]. In San Francisco, $4.5 million of the expected $10 million revenue from sugar sweetened beverage taxes will fund grants supporting community-based organizations and $2.5 million will be given to the San Francisco Unified School District to provide healthy meals, water fountain maintenance and oral care in schools [14].

The second factor is whether advocates can provide an effective response to arguments against taxes. One promising approach is for supporters of taxes to acknowledge that there is a trade-off between individuals’ access to marketed products and the community’s ability to take action against significant threats to health. This view argues that as with cigarette taxes, modest restrictions of individual choice are justifiable when they support individual and community health and well-being.

This argument can be extended into a response to concerns that the taxes are unfair to low-income families. Tobacco taxes also disproportionately burden those with limited income. However, in part because such taxes also disproportionately reduce smoking and the burden of tobacco-related diseases in low-income communities, such taxes have been broadly accepted. More controversial are regressive taxes on necessities, such as groceries, with the majority of U.S. states exempting such products from general sales taxes. Thus the perceived fairness of a SSB tax may depend on whether SSBs are seen more like tobacco products (harmful, not necessities) or more like groceries (necessities). The heavy burden of diabetes and obesity in lower socioeconomic households, along with the evidence linking SSB and unhealthy foods with these diseases supports the need for a policy level intervention, such as a tax, to improve the health of these very communities. Resolving difficult questions of equity requires engagement with communities that would be most affected by the policy in policy discussions, to assess their preferences and priorities. Policymakers should consider earmarking funds from taxes on unhealthy foods specifically to benefit lower socioeconomic communities.

The pace of change in responding to obesity reflects both the relative strength of the evidence and the changing acceptability of various options. Given the difficulty in changing diets and reducing obesity, the lack of “easy” solutions, and the potential for a convincing case to be made for adoption, there is every reason for communities to consider taxation of unhealthy beverages and foods as part of a comprehensive approach to diet and health.
